# Redox processes are major regulators of leukotriene synthesis in neutrophils exposed to bacteria *Salmonella typhimurium*; the way to manipulate neutrophil swarming

**DOI:** 10.3389/fimmu.2024.1295150

**Published:** 2024-02-07

**Authors:** Ekaterina A. Golenkina, Galina M. Viryasova, Svetlana I. Galkina, Natalia D. Kondratenko, Tatjana V. Gaponova, Yulia M. Romanova, Konstantin G. Lyamzaev, Boris V. Chernyak, Galina F. Sud’ina

**Affiliations:** ^1^ Belozersky Institute of Physico-Chemical Biology, Lomonosov Moscow State University, Moscow, Russia; ^2^ National Research Center for Hematology, Russia Federation Ministry of Public Health, Moscow, Russia; ^3^ Department of Genetics and Molecular Biology, Gamaleya National Research Centre of Epidemiology and Microbiology, Moscow, Russia; ^4^ The “Russian Clinical Research Center for Gerontology” of the Ministry of Healthcare of the Russian Federation, Pirogov Russian National Research Medical University, Moscow, Russia

**Keywords:** neutrophil, *Salmonella typhimurium*, leukotriene B4, reactive oxygen species, glutathione, neutrophil swarming

## Abstract

Neutrophils play a primary role in protecting our body from pathogens. When confronted with invading bacteria, neutrophils begin to produce leukotriene B4, a potent chemoattractant that, in cooperation with the primary bacterial chemoattractant fMLP, stimulates the formation of swarms of neutrophils surrounding pathogens. Here we describe a complex redox regulation that either stimulates or inhibits fMLP-induced leukotriene synthesis in an experimental model of neutrophils interacting with *Salmonella typhimurium*. The scavenging of mitochondrial reactive oxygen species by mitochondria-targeted antioxidants MitoQ and SkQ1, as well as inhibition of their production by mitochondrial inhibitors, inhibit the synthesis of leukotrienes regardless of the cessation of oxidative phosphorylation. On the contrary, antioxidants N-acetylcysteine and sodium hydrosulfide promoting reductive shift in the reversible thiol-disulfide system stimulate the synthesis of leukotrienes. Diamide that oxidizes glutathione at high concentrations inhibits leukotriene synthesis, and the glutathione precursor S-adenosyl-L-methionine prevents this inhibition. Diamide-dependent inhibition is also prevented by diphenyleneiodonium, presumably through inhibition of NADPH oxidase and NADPH accumulation. Thus, during bacterial infection, maintaining the reduced state of glutathione in neutrophils plays a decisive role in the synthesis of leukotriene B4. Suppression of excess leukotriene synthesis is an effective strategy for treating various inflammatory pathologies. Our data suggest that the use of mitochondria-targeted antioxidants may be promising for this purpose, whereas known thiol-based antioxidants, such as N-acetylcysteine, may dangerously stimulate leukotriene synthesis by neutrophils during severe pathogenic infection.

## 1 Introduction

The main effector functions of polymorphonuclear leukocytes (PMNLs, neutrophils) in the fight against pathogens include phagocytosis, oxidative burst, degranulation. They eliminate pathogens through the production of reactive oxygen species (ROS) and releasing azurophilic granules containing antimicrobial proteins such as neutrophil elastase and myeloperoxidase (MPO) (1, 2). Nitric oxide (NO) production in human PMNLs, along with ROS and MPO is important to execute antimicrobial activity (3). In cases where these weapons are not effective enough to kill pathogens, a program of collective behavior known as swarming can be initiated. This program involves the production of leukotriene B4 (LTB4) as a potent chemoattractant and the gathering of neutrophils into dense clusters surrounding pathogens (4). In these clusters, neutrophils activate suicidal production of extracellular chromatin traps (NETs), which enhance pathogen fixation (5). Both swarming and NETs formation (NETosis) programs are subject to complex redox regulation.

Early neutrophil recruitment is initiated by pathogen-associated molecular patterns (PAMPs), including N-formyl peptides, and damage-associated molecular patterns (DAMPs) (6). This interaction of “pioneer” neutrophils with pathogen (or danger signal from damaged tissue) results in leukotriene B4 (LTB4) synthesis, and started the next step, swam attractant release (7), leading to exponential accumulation of neutrophils at infection/damage loci (8). At this stage, LTB4 and the receptors BLT1 for LTB4 coordinate cellular responses by neutrophils with each other, and the swarm is formed. Also, the coordinated transcellular biosynthesis of LTB4 drives swarming responses (9). The initiation of swarming converges on the synthesis of LTB4. During this swarm recruitment NETs could not be observed. On the timeline of major events in neutrophil swarming, the onset of NETs forming proceeds later, on the aggregation phase of the swarming response when neutrophils clusters surrounding pathogens has been formed, and activated cells release chromatin, which is accompanied with the loss of integrity of cellular membrane (5, 10).

Leukotrienes play role also in inhibition of neutrophil swarming (6). ω-OH-LTB4 and ω-COOH-LTB4 compete with LTB4 for BLT1 receptor binding (11) and act as inhibitors of LTB4-mediated responses. When LTB4 is easy transformed to ω-OH-LTB4, this decreases neutrophil swarming. We recently found that stimulation by fMLP of neutrophils after preincubation with bacteria *Salmonella typhimurium* strongly increased leukotriene synthesis; but when the bacteria:neutrophil ratio increased, the transformation of LTB4 to ω-OH-LTB4 was suppressed (12), which support increased level of LTB4. LTB4 is the strongest chemoattractant and works at sub nanomolar concentrations (13). The increased formation of LTB4 during the interaction of neutrophils with bacteria works as a signal of neutrophils for help with an increase in bacterial load. In this study we explored intervention of redox processes in neutrophil on fMLP-induced leukotriene synthesis in the experimental model of neutrophil interaction with bacteria *Salmonella typhimurium*.

NADPH-oxidase (NOX2) is the primary source of ROS which is not only responsible for oxidative burst but also involved in phagocytosis (14), degranulation (15) and NETosis (16). ROS were required for PMNLs antimicrobial activity against *S. pneumoniae*; however the NADPH oxidase was dispensable for that (17). *S. pneumoniae* infection induced mitochondrial ROS production in PMNLs. And mitochondrial ROS were critical for the ability of PMNLs to kill *S.pneumoniae*. DPI which inhibits ROS production by the NADPH oxidase, did not blunt the ability of PMNs to kill *S. pneumoniae*, but MitoTempo did. Dunham-Snary et al. (18) were first who showed that neutrophil mitochondria actively participate in phagocytosis and killing of *Staphylococcus aureus*, and antimycin and MitoTempo increased bacterial survival (18). Human pathogen *Shigella* dramatically changed neutrophils toward enhanced microbial recognition and mitochondrial ROS production (19). What is the role of mitochondrial ROS in leukotriene synthesis in infected neutrophils?

Mitochondria are an important source of ROS in various cell types, but their role in ROS production in neutrophils has long been underestimated. Only our recent studies (20–22) using the mitochondria-targeted antioxidant SkQ1 [10-(6’-plastoquinonyl)decyltriphenylphosphonium bromide] (23) have demonstrated the important role of mitochondrial ROS (mtROS) in NADPH oxidase activation, degranulation, extracellular trap formation (NETosis), and leukotriene synthesis. These studies analyzed the activation of neutrophils by the Ca^2+^ ionophore A23187 and the chemoattractant N-formyl-L-methionyl-L-leucyl-L-phenylalanine (fMLP), while the role of mtROS in the interaction of neutrophils with bacteria remains unknown.

Neutrophils control the infection, in turn, microorganisms affect the functions of neutrophils, controlling phagocytosis, the production of oxidants and the lifespan of neutrophils (24). Pathogens antagonize neutrophils, for example, by secreting catalase to reduce ROS (25, 26). To protect from ROS, the fungal pathogen *Histoplasma capsulatum* infects both neutrophils and macrophages producing a superoxide dismutase (SOD) (27). Gram-negative pathogen *Coxiella burnetii* (28) and *Pseudomonas aeruginosa* (26), as well as *E.coli* producing enterobactin (29) inhibit NADPH oxidase in human neutrophils. Microbial avoidance strategies can target not only ROS production but also degranulation and synthesis of leukotriene B4 (30). Decreased intracellular GSH correlates with the susceptibility to infections. Glutathione reductase (Gsr) catalyzes the reduction of glutathione disulfide to glutathione using NADPH as an electron donor (31). Glutathione reductase promotes *Candida albicans* clearance (32). As NADPH is a cofactor required for GSH regeneration from GSSG, the consumption of NADPH affects the regeneration of GSH (33). One can propose that NADPH-oxidase inhibition can support GSH level. These processes certainly affect LT synthesis and neutrophil swarming around pathogens.

5-Lipoxygenase (5-LOX) is a key enzyme in synthesis of LTB4 involved in chemical cell-to-cell communication. LTB4 is critical for enhancing chemotactic responses to primary chemoattractants, such as fMLP (34) increasing clustering and surface mobility of adhesion receptors integrins (35). Thus, LTB4 is not only a chemotactic, but also a neutrophil aggregating substance (36) that promotes local neutrophil interactions during swarming (4). Initial neutrophil–neutrophil contacts are critical to initiate swarming (37). We recently found, that the synthesis of LTB4 in neutrophils in the presence of bacteria and fMLP correlates with the appearance of cell-cell contacts (12), which can serve as a signal conductor to further clustering and swarming.

In the current study, we observed that redox processes can either activate or inhibit fMLP-induced leukotriene synthesis in an experimental model of neutrophil interaction with the bacteria *Salmonella typhimurium*. Our study demonstrated that mitochondrial ROS are crucial for 5-LOX activation and LT synthesis, and mitochondria-targeted antioxidants inhibited LT synthesis. On the other hand, inhibition of NOX2-dependent ROS supported LTB4 synthesis, and potentiated the stimulating effect of thiol oxidant diamide on LT synthesis.

## 2 Materials and methods

Hank’s balanced salt solution with calcium and magnesium but without Phenol Red and sodium hydrogen carbonate (HBSS), Dulbecco’s phosphate-buffered saline (PBS) with magnesium but without calcium, N-Formyl-L-methionyl-L-Leucyl-L-Phenylalanine (fMLP), Ellman’s reagent [5,5′-Dithiobis(2-nitrobenzoic acid)], oxythiamine, and fibrinogen from human plasma, were purchased from Sigma (Steinheim, Germany). Dextran T-500 was from Pharmacosmos (Holbæk, Denmark). ROS indicator Carboxy-H_2_DCFDA and Pierce™ Avidin, Fluorescein (FITC) conjugated were from Thermo Fisher Scientific (Waltham, MA USA). Mitochondrial ToxGlo™ Assay was from Promega Corp. (Madison, WI USA). Biotinylated murine IgG1 antibodies CD11b and CD54 were from Ancell Corp. (Bayport, MN USA). Bacteria (*S. typhimurium* IE 147 strain) were obtained from the Collection of Gamaleya National Research Center of Epidemiology and Microbiology (Moscow, Russia). Bacteria were grown in Luria–Bertani broth to a concentration of 1 × 10^9^ colony-forming units (CFU)/mL. In this study not opsonized bacteria were used.

### 2.1 Neutrophil isolation

Human polymorphonuclear leukocytes (PMNL) were isolated from freshly collected blood with citrate anticoagulant. Leukocyte-rich plasma was obtained from donated blood by sedimentation in the presence of dextran T-500. Granulocytes were obtained as described (38). Cell viability was checked by trypan blue exclusion. Control suspension samples were stained in parallel with Hoechst and Romanovsky-Giemsa dyes to assess the homogeneity of the cell population. PMNLs (95–97% purity, 98–99% viability) were stored at room temperature in Dulbecco’s PBS containing 1 mg/mL glucose (no CaCl2) until use.

### 2.2 Determination of 5-LOX product formation in cells

PMNLs [(1.2-1.5)x10^7^/6 ml HBSS with 10 mM HEPES (HBSS/HEPES)] were pre-incubated for 10 min at 37°C, 5% CO_2_. At this stage, 2-deoxy-D-glucose (2-DG) was added to the samples, in those cases where this was provided for in the experimental protocol. Then, maintaining incubation conditions, *S. typhimurium* (bacteria per cell ratio ~25:1) and indicated reagents were added for 30 min, followed by 10 min exposure to 0,1 µM fMLP. The treatment was stopped by adding of an equal volume of methanol (-18°C) with 90 ng PGB2 as internal standard. Major 5-LOX metabolites, 5S, 12R-dihydroxy-6,14-*cis*-8,10-*trans*-eicosatetraenoic acid (LTB4), iso-LTB4 (5S, 12SR-all-*trans*-diHETE), ω-OH-LTB4, ω-COOH-LTB4 and 5S-hydroxy-6-*trans*-8,11,14-*cis*-eicosatetraenoic acid (5-HETE) were identified as previously described (12).

### 2.3 ATP assessment

ATP detection component from Mitochondrial ToxGlo™ Assay kit was used. In accordance with the manufacturer’s protocol, the lyophilized enzyme/substrate mixture (ATP Detection Substrate) was reconstituted by lysis buffer (ATP Detection Buffer) to obtain ATP Detection Reagent. Just before the experiment PMNLs were resuspend in HBSS/HEPES, seeded in solid white F-bottom 96-well plates (4 × 10^5^ cells/well), pre-incubated for 10 min at 37°C, 5% CO_2_. 2-DG was added at this stage if prescribed by the protocol. Then *S. typhimurium* (bacteria per cell ratio ~25:1) and reagents were added, according to experimental protocol. Samples were incubated for 20 min under the same conditions. After the treatment was complete, plates were equilibrated to room temperature for 5 min followed by adding an equal volume of ATP Detection Reagent to the contents of each well. After 3 min orbital shaking luminescence intensity was measured on a CLARIOstar microplate reader (BMG Labtech, Ortenberg, Germany) and MARS data analysis software package from BMG Labtech was used to process the data obtained.

### 2.4 Cytosolic ROS assessment

ROS accumulation in the cytosol was quantified by measuring the green fluorescence intensity of 2’,7’-dichlorofluorescein (DCF). According to manufacturer’s recommendation, neutrophils were loaded with 5 μM carboxy-2’,7’-dichlorodihydrofluorescein diacetate (H_2_DCF-DA) for 60 min at room temperature followed by washing with PBS, suspended in D-PBS and then stored at room temperature in the dark until use. Immediately before the experimental treatment cells were resuspended in HBSS/HEPES, seeded in fibrinogen-coated wells of the 96-well plate (4 × 10^5^ cells/well) and pre-incubated for 10 min at 37°C, 5% CO_2_. Then *S. typhimurium* (bacteria per cell ratio ~25:1) and indicated reagents were added for 30 min followed by 0,1 µM fMLP stimulation. Fluorescence intensity at excitation and emission wavelengths of 488 and 525 nm was measured using a CLARIOstar microplate reader.

### 2.5 Thiol redox state assessment

Ellman’s assay was used for quantitating reduced sulfhydryl groups. Neutrophils were resuspended in HBSS/HEPES (2 × 10^6^ cells/1 mL probe) and pre-incubated for 10 min at 37°C, 5% CO_2_. After the treating according to experimental protocol cells were centrifuged for 7 min at 600 g, 4°C. Permeabilizing buffer (67 mM Na_2_HPO_4_, 35 mM citric acid and 0.1% Triton X-100; 100 µL/probe) was added to packed cell pellets, shaken and kept on ice for 10 min. Lysates were centrifuged at 10000 g, 4°C for 10 min. 50 µL supernatant (in duplicate for each probe) was mixed with 100 µL DTNB solution (0.1 mg/mL in 0.1 M NaH_2_PO_4_/Na_2_HPO_4_, 1M EDTA, pH 7.8) in 96-well plate. Equal volumes of reduced glutathione solutions from 1 to 500 µM were used to generate standard curve. The samples were allowed to stand for 15 min at room temperature followed by absorbance reading at 412 nm on CLARIOstar microplate reader.

### 2.6 Adhesion assessment

Spectrophotometric detection of 2,3-diaminophenazine, which is a product of myeloperoxidase-catalyzed oxidation of *o*-phenylenediamine dihydrochloride (OPD) by hydrogen peroxide, was used to assess neutrophil substrate adhesion (39). PMNLs (2 × 10^5^ cells/sample) were seeded onto fibrinogen-coated 96-well plates containing pre-warmed HBSS/HEPES and agents required by experimental protocol. Samples were incubated at 37°C, 5% CO_2_ after which the plate was washed twice to remove unattached cells. 4 mM H_2_O_2_ and 5.5 mM OPD in permeabilizing buffer (67 mM Na_2_HPO_4_, 35 mM citric acid and 0.1% Triton X-100) were added for 5 min. The reaction was stopped with 1 M H_2_SO_4_. The percentage of attached neutrophils was determined by measuring the absorption (490 nm) of 2,3-diaminophenazine and comparing the obtained values with the calibration ones.

### 2.7 Scanning electron microscopy

For scanning electron microscopy, cells were fixed for 30 min in 2.5% glutaraldehyde, postfixed for 15 min with 1% osmium tetroxide in 0.1 M cacodylate (pH 7.3), dehydrated in an acetone series, and processed by conventional scanning electron microscopic techniques, as described (40).

### 2.8 Cell adhesion molecules expression assessment

CD11b, alpha subunit of the Mac-1 integrin, and intercellular adhesion molecule-1 (CD54) proteins expression were determined by flow cytometry. Neutrophils were resuspended in HBSS/HEPES (10^6^ cells/1 mL probe) and pre-incubated for 5 min at 37°C, 5% CO_2_. After the treating according to experimental protocol cells were centrifuged while cooled for 10 min at 200 g, 4°C. Then biotinylated mouse CD11b (20 µg/mL HBSS/HEPES) or CD54 (30 µg/mL HBSS/HEPES) antibodies were added for 45 min, on ice. After being washed with cold PBS cells were stained with avidin-FITC (30 µg/mL HBSS/HEPES) for 30 min on ice followed by flow cytometry on Amnis FlowSight Imaging Flow Cytometer (Luminex Corp., Austin, Texas, USA) at 488/525 ex/em filter set. IDEAS Image Data Exploration and Analysis Software (Luminex Corp., ustin, Texas, USA) was used for data analyzing.

### 2.9 Statistics

Results are presented as mean ± SEM. Analysis of statistical significance for multiple comparisons was performed using GraphPad Prism 10.0.1 software. Differences with P-values <0.05 were considered statistically significant.

## 3 Results

### 3.1 Mitochondrial ROS production is critical for fMLP-induced leukotriene synthesis induced in neutrophils stimulated with *Salmonella typhimurium*

We have previously found that preincubation of human neutrophils with *S. typhimurium* strongly stimulates fMLP-induced LTB4 synthesis (12). These conditions modeled the collective behavior of neutrophils known as swarming (4). We have shown that *S. typhimurium* stimulates 5LOX, but the mechanism of the enhancing effect of bacteria upon subsequent addition of fMLP remains unclear. We have recently shown that LTB4 synthesis induced by fMLP (as well as some other stimuli) is dependent on mtROS production (22). However, the effect of fMLP in this study was artificially stimulated by cytochalasin, which disrupts the actin cytoskeleton. In the present study, we analyzed the possible role of mtROS in the stimulation of fMLP-induced LTB4 synthesis in neutrophil interaction with bacteria *S. typhimurium*. Separate addition of fMLP (Control w/o Salm) or bacteria *S. typhimurium* (Control w/o fMLP) to neutrophils produced very slight effect on leukotriene synthesis (**Supplementary Figure 1**).

**Figure 1:**
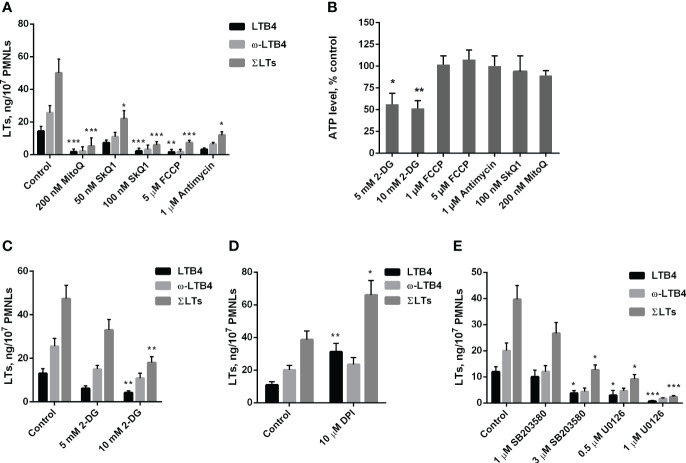
**(A–C)**. Effect of mitochondria-targeted compounds and 2-DG treatment on leukotriene synthesis and ATP generation in neutrophils. **(A, C)** After 10 min pre-incubation without **(A)** or in the presence of indicated concentrations of 2-DG **(C)** PMNLs were exposed for 30 min to either bacteria alone (Control) or bacteria in combination with reagents indicated on X-axis. Then fMLP was added (0.1 µM sample concentration) for 10 min. After the reaction was terminated, the 5-LOX products were analyzed. Presented are absolute values of LTB4, ω-OH-LTB4 and the sum of LTs (ΣLTs) in ng per 10^7^ PMNLs. **(B)** PMNLs were pre-incubated for 10 min without additives or in the presence of 2-DG (where indicated). Bacteria were then added, either alone (Control, 2-DG probes) or in combination with the indicated stimuli. After 20 min, cells were lysed, and ATP content was determined using the bioluminescent method. Presented are relative values of ATP content in samples as average percentages of control luminescence intensity values (mean ± SEM of luminescence intensity in control samples was 105326 ± 4596 RLU, while 10 µM ATP, used as a positive control, provided 397233± 2187 RLU). **(D, E)**. Effect of NADPH oxidase inhibitors and inhibitors of MAP kinases on leukotriene synthesis in neutrophils. After 10 min pre-incubation, PMNLs were exposed for 30 min to either bacteria alone (Control) or bacteria in combination with reagents indicated on X-axis, followed by 10 min of 0.1 µM fMLP stimulation. When incubations stopped, the 5-LOX products were analyzed. **(A–E)**. Values shown are means ± SEM of three independent experiments performed in duplicate. *p < 0.05, **p < 0.01, ***p < 0.001, for pairs of data compared to corresponding control values as shown by two-way ANOVA with Tukey’s multiple comparison test **(A, C–E)** or by one-way ANOVA with Dunnett’s multiple comparison test **(B)**.

As shown in **Figure 1A**, mitochondria-targeted antioxidants SkQ1 and MitoQ inhibit fMLP-induced LTB4 synthesis in neutrophils exposed to *S. typhimurium* at 50 nM and 200 nM, respectively. The synthesis of the omega-hydroxylation product of LTB4 (ω-LTB4) and total leukotrienes (ΣLT=LTB4+isomers of LTB4+ω-OH-LTB4) were also inhibited by SkQ1 and MitoQ. These leukotrienes are the main 5-LOX products in our experimental model (**Supplementary Figure 1**).

We have previously shown that mtROS production in neutrophils can be stimulated by the accumulation of Ca^2+^ in mitochondria (21). Ca^2+^ uptake into the mitochondrial matrix is driven by the transmembrane electrical potential at the inner membrane (ΔΨ), which is maintained by the activity of the respiratory chain. In the present model, the respiration inhibitor antimycin A, as well as the oxidative phosphorylation uncoupler FCCP, which dissipates ΔΨ, effectively inhibited leukotriene synthesis (**Figure 1A**). Inhibition of respiration and dissipation of ΔΨ lead to the cessation of mitochondrial ATP synthesis. Since mitochondria-targeted cationic antioxidants such as SkQ1 and MitoQ have also been shown to dissipate ΔΨ (41), we analyzed the possible role of ATP depletion in the inhibition of leukotriene synthesis. Measurements of ATP content in infected neutrophils revealed no effect of antimycin A or FCCP (**Figure 1B**). These data are consistent with the leading role of glycolysis in ATP production known for neutrophils and other granulocytes (42, 43). In support of this conclusion, inhibition of glycolysis by 2-deoxy-D-glucose (2-DG) led to a decrease in ATP content (**Figure 1B**) and inhibition of leukotriene synthesis (**Figure 1C**).

**Figure 2:**
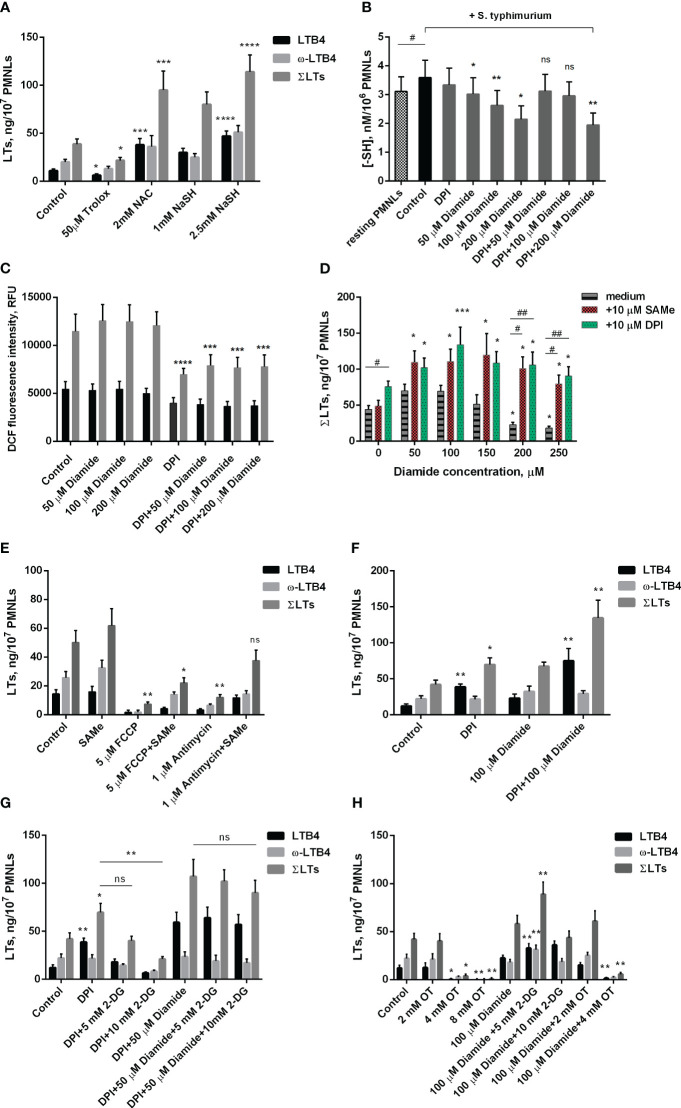
Effect of oxidants and antioxidants on intracellular reduced thiols level, ROS formation and leukotriene synthesis in human neutrophils. **(A, D-H)**. After 10 min pre-incubation without additives or in the presence of indicated concentrations of 2-DG, PMNLs were exposed for 30 min to either bacteria alone (Control) or bacteria in combination with reagents indicated (10 µM DPI and 10 µM SAMe were used), followed by 10 min of 0.1 µM fMLP stimulation. After the reaction was terminated, the 5-LOX products were analyzed. Presented are absolute values of LTB4, ω-OH-LTB4 and the sum of LTs (ΣLTs) in ng per 10^7^ PMNLs. **(B)** PMNLs were pre-incubated for 10 min, then *S. typhimurium* alone (Control) or bacteria together with indicated stimuli were added for 20 min; resting PMNLs samples were incubated without any additives. After the exposure time, the cells were pelleted while cooling and assayed for reduced -SH content with Ellman’s reagent. Values are given as the averages of the content of reduced thiols in the samples, nM per 10^6^ PMNLs. **(C)** PMNLs loaded with H_2_DCFDA were pre-incubated for 10 min, then either *S. typhimurium* alone (Control) or bacteria together with indicated stimuli were added. After 30 min 0,1 µM fMLP was added. Presented are the average values of DCF fluorescence intensity measured immediately before (black bars) and 20 minutes after (grey bars) fMLP addition. **(A–H)**. Values shown are means ± SEM of three independent experiments performed in duplicate. ns, not significant; *p < 0.05, **p < 0.01, ***p < 0.001, ****p < 0.0001 for pairs of data compared to corresponding control values; ^#^p < 0.05, ^##^p < 0.01 for the specified data pairs as shown by one-way ANOVA with Dunnett’s multiple comparison test **(B)** or two-way ANOVA followed by Tukey’s multiple comparison test **(A, C–H)**.

Stimulation of neutrophils by bacteria and fMLP is accompanied by activation of NADPH oxidase (NOX2) and subsequent massive production of ROS (1, 44). To study the possible role of NOX2 in leukotriene synthesis in our model, we used diphenyleneiodonium (DPI), an effective inhibitor of various flavin enzymes, including NADPH oxidase. DPI stimulated the accumulation of LTB4 and total leukotrienes (**Figure 1D**). This effect was observed previously when leukotriene synthesis was induced by various stimuli and was attributed to inhibition of LTB4 omega-hydroxylation (22). In fact, the ratio of LTB4 to ω-LTB4 with DPI is higher, than in other treatments (**Figures 1**, **2**). But the sum of LTs is also increased, therefore inhibition of NOX2 contribute to stimulation of leukotriene synthesis. It was demonstrated that chronic granulomatous disease (CGD) neutrophils have an increased LTB4 production (45). But the authors determined only LTB4, so this effect may include as 5-LOX stimulation by downregulation of ROS in CGD neutrophils, as well as inhibition of LTB4 omega-hydroxylation in experiments with DPI.

From these data we can see that mitochondria targeted antioxidant SkQ1 inhibits LT synthesis. We checked the effects of SkQ1 on mtROS production in neutrophils. Measurements of mitochondrial ROS production using the mitochondria-targeted superoxide-sensitive probe MitoSOX (**Supplementary Figure 2**) showed a significant increase in mtROS upon subsequent stimulation of neutrophils with *S. typhimurium* and fMLP. This increase in mtROS was inhibited by SkQ1. These data support our conclusion that mtROS production is required for stimulation of fMLP-induced LTB4 synthesis in neutrophil interaction with bacteria *S. typhimurium*.

In the search for possible targets of mtROS-dependent regulation of leukotriene synthesis, we previously showed that activation of the MAP kinases Erk1/2 and p38 (associated with their phosphorylation) is prevented by SkQ1 (22). Erk1/2 and p38 kinases are known to phosphorylate 5-LOX and stimulate its translocation to the nuclear envelope, which is required for 5-lipoxygenase activity (46), so they may be critical targets of mtROS. In our experimental model, the Erk1/2 inhibitor U0126 and p38 inhibitor SB203580 suppressed 5-LOX activity (**Figure 1E**). Thus, it can be assumed that these kinases are important in the mtROS-dependent regulation of leukotriene synthesis induced by the combined action of bacteria and fMLP.

### 3.2 Redox processes modulate fMLP-induced leukotriene synthesis in neutrophils exposed to bacteria *Salmonella typhimurium*

To further study the role of the redox balance in the activation of leukotriene synthesis under the combined action of *S. typhimurium* and fMLP, we used the classical antioxidant Trolox, a water-soluble analogue of vitamin E. It was shown that it inhibits leukotriene synthesis, although at a much higher concentration than SkQ1 and MitoQ (**Figure 2A**).

Unexpectedly, another known antioxidant N-acetylcysteine (NAC) strongly stimulated fMLP-induced leukotriene synthesis in *S. typhimurium*-activated neutrophils (**Figure 2A**). A similar effect was observed in the case of sodium hydrosulfide (NaSH) (**Figure 2A**), an H_2_S donor that promotes a reductive shift in the redox balance of various cells (47). We hypothesized that this stimulation may be associated with a shift in the redox equilibrium of the reversible thiol-disulfide system and, first of all, with an increase in the ratio of reduced/oxidized glutathione (GSH/GSSG). In fact, NAC is a direct precursor of GSH, and H_2_S donors can also increase the GSH/GSSG ratio. To analyze this possibility, we used diamide, which penetrates cell membranes and reacts with thiols to form disulfides (48). Diamide at a concentration of 50-200 μM significantly reduced the level of non-protein thiols (**Figure 2B**) but did not affect the level of cytosolic ROS measured as the oxidation of 2’,7’-dichlorofluorescein (**Figure 2C**). 50-150 μM diamide slightly increased fMLP-induced leukotriene synthesis in neutrophils pre-incubated with *S. typhimurium*, while 200-250 μM diamide, without affecting cell viability (**Supplementary Figure 3**), significantly inhibited leukotriene synthesis (**Figure 2D**). These data indicate that a decrease in reduced GSH may limit leukotriene synthesis under our conditions. In support of this assumption, we observed a strong stimulation of leukotriene synthesis by the GSH precursor S-adenosyl-L-methionine (SAMe) (**Figure 2D**). This effect was especially strong in the presence of an inhibitory concentration of diamide (200-250 μM) and reaches approximately 400%. Interestingly, SAMe also reversed inhibitory effect of FCCP or antimycin A on leukotriene synthesis (**Figure 2E**). Although in our experimental model we were unable to detect a significant effect of antimycin A and FCCP on GSH/GSSH ratio either at the stage of PMNLs interaction with bacteria or after stimulation with fMLP (**Supplementary Figure 4**), studies on cell lines indicate that they both can reduce GSH intracellular level (49–51), which can be compensated by SAMe. Measurements of mtROS in stimulated neutrophils (**Supplementary Figure 2**) showed some increase by diamide, which may be partially responsible for the stimulation of LT synthesis observed at low doses of diamide.

As shown in **Figure 2B**, under conditions of interaction between neutrophils and bacteria, 50 to 200 μM diamide reduced intracellular -SH amount. The decrease in reduced thiol content caused at 50 and 100 μM diamide was prevented by DPI. Under the same conditions DPI decreased the level of cytosolic ROS, presumably due to inhibition of NOX2, and diamide did not modulate this effect (**Figure 2C**). These data suggest that the decrease in GSH/GSSG ratio by low doses of diamide may be compensated by the decrease in oxidative stress and increase in NADPH levels caused by inhibition of NADPH oxidase. This effect may explain the DPI stimulation of leukotriene synthesis (**Figure 1D**), which is especially pronounced in the presence of diamide (**Figure 2D**), with maximal stimulation at 100 μM diamide (**Figure 2F**). DPI did not reverse the decrease in reduced thiols at 200 μM diamide but abolished inhibiting effect of diamide on LT synthesis at 200 μM (**Figure 2D**). We can only propose that effect of DPI is more complex than we determined and need further elucidation.

Biosynthesis of GSH is dependent on ATP, so inhibition of leukotriene synthesis due to 2-DG-dependent ATP depletion (**Figure 1B**) may be mediated by a decrease in GSH content. 2-DG also inhibits the synthesis of leukotrienes stimulated by DPI alone (**Figure 2G**). Interestingly, the synthesis of leukotrienes stimulated by DPI in the presence of diamide is practically insensitive to 2-DG (**Figure 2G**). This may be explained by the known ability of diamide to reduce glucose metabolism through glycolysis in favor of the pentose phosphate pathway (PPP) (52). This pathway is the main source of NADPH (42), which supports the reduction of glutathione. This effect probably underlies the stimulation of leukotriene synthesis observed at low doses of diamide (**Figure 2D**).

Full pentose cycle, including non-oxidative PPP with enzyme transketolase (TKT), provides maximal NADPH yield (6 NADPH from one molecule of glucose) (53). In our assay TKT inhibitor oxythiamine (OT) suppressed LT synthesis (**Figure 2H**). The effect was observed with and without diamide in incubations. It is known that blockage of glycolysis can help cells divert more flux into oxPPP under oxidative stress (54, 55). With diamide, LT synthesis was increased in the presence of intermediate concentrations of 2-DG (**Figure 2H**), and not in control incubations (**Figure 1C**). We did not observe it in the presence of DPI (**Figure 2G**).

**Figure 3:**
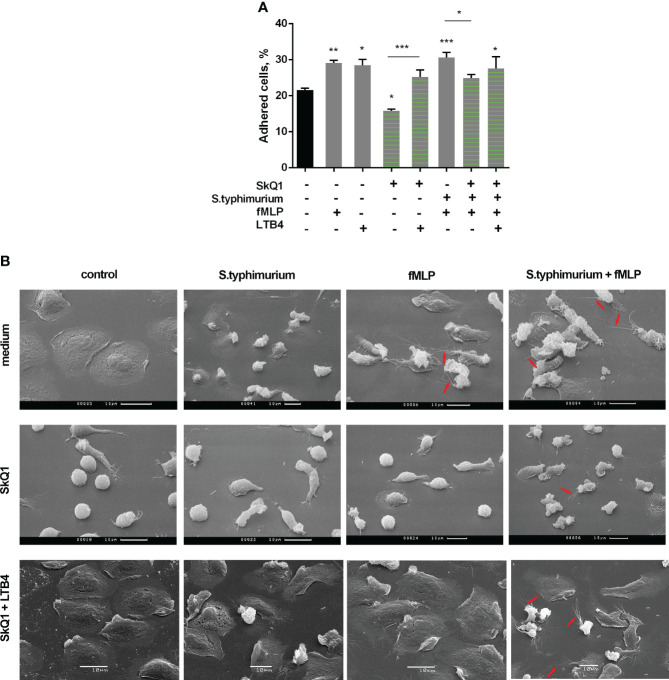
**(A)** SkQ1 influence on the pro-adhesive effects of fMLP and LTB4. PMNLs (2 × 10^5^ cells/sample) were seeded onto fibrinogen-coated 96-well plates containing pre-warmed HBSS/HEPES without additives, with *S. typhimurium* (bacteria per cell ratio ~20:1) or *S. typhimurium* supplemented with 0.1 µM SkQ1. After 30 min incubation at 37°C, 5% CO_2_, 0.1 µM fMLP or/and 0.1 µM LTB4 were added for next 10min. Values shown means ± SEM of the percentage of cells attached to the substrate obtained from three independent experiments performed in triplicates; *p < 0.05, **p < 0.01, ***p < 0.001 for indicated pairs of data or compared to resting PMNLs sample (black bar) as shown by ordinary one-way ANOVA with Sidak’s multiple comparison test. **(B)** Effect of SkQ1 and LTB4 on neutrophil morphology upon (co-)stimulation with bacteria *S. typhimurium* and fMLP. PMNLs (10^6^/ml HBSS/HEPES) were preincubated on coverslips in culture dishes for 10 min and then cultured for 20 min without additives (line *medium*) or in the presence of 100 nM SkQ1 (lines *SkQ1*, *SkQ1+LTB4*) without additional stimulation (control) or with the addition of bacteria (bacteria per cell ratio ~25:1) (S. typhimurium). LTB4 (100 nM sample concentration) was added to (control) and (S. typhimurium) samples for next 5 minutes (line *SkQ1+LTB4*). Columns fMLP and S. typhimurium+fMLP represent PNMLs incubated for 20 min without additives (line *medium*) or with 100 nM SkQ1 (lines *SkQ1*, *SkQ1+LTB4*) without bacteria (fMLP) or with the addition of bacteria (S. typhimurium+fMLP), followed by fMLP (0.1 µM sample concentration) (lines *medium*, *SkQ1*) or fMLP together with LTB4 (100 nM sample concentration of each) (line *SkQ1+LTB4*) treatment for 5 minutes. At the end of each stage, PMNLs samples were fixed and subsequently visualized using scanning electron microscopy. Thread-like cell outgrowths are marked by red arrows.

Under the influence of fMLP, neutrophil adhesion is significantly enhanced (**Figure 3A**). In addition, this chemotactic peptide induces noticeable morphological changes in both resting and interacting with bacteria neutrophils. In addition to pronounced cellular polarization, special mention should be made to the formation of numerous thread-like cell outgrowths (**Figure 3B**, control/fMLP, S.typhimurium+fMLP, red arrows). It is known that such outgrowths are a kind of “transport highways” that allow intercellular exchange over long distances (56), and also perform a structural function, promoting cell clustering (57). Mitochondria-targeted antioxidant SkQ1 significantly reduced the substrate adhesion of neutrophils (**Figure 3A**). Preincubation with SkQ1 not only reduce the pro-adhesive effect of fMLP but also prevents fMLP-induced morphological changes, in particular, it diminishes the formation of cell outgrowths (**Figure 3B**), in parallel with suppression of leukotriene synthesis (**Figure 1A**). The addition of LTB4 to samples pretreated with SkQ1 increased the adhesiveness of neutrophils, promoting cell spreading (**Figure 3**, SkQ1+LTB4). In addition, supplementation of fMLP influence on cells preincubated with SkQ1 and bacteria with exogenous leukotriene B4 restored the ability of neutrophils to form thread-like filaments (**Figure 3**, SkQ1+LTB4/S.typhimurium+fMLP, red arrows). These data support our earlier suggestion that the emergence of cell-cell contacts is dependent on LTB4 (12). 50 µM diamide also increased adhesiveness of neutrophils (**Supplementary Figure 5A**). At high diamide concentration, when leukotriene synthesis was inhibited, the cells change morphology to more spherical shape, and the adhesiveness of neutrophils decreased (**Supplementary Figure 5**).

**Figure 4:**
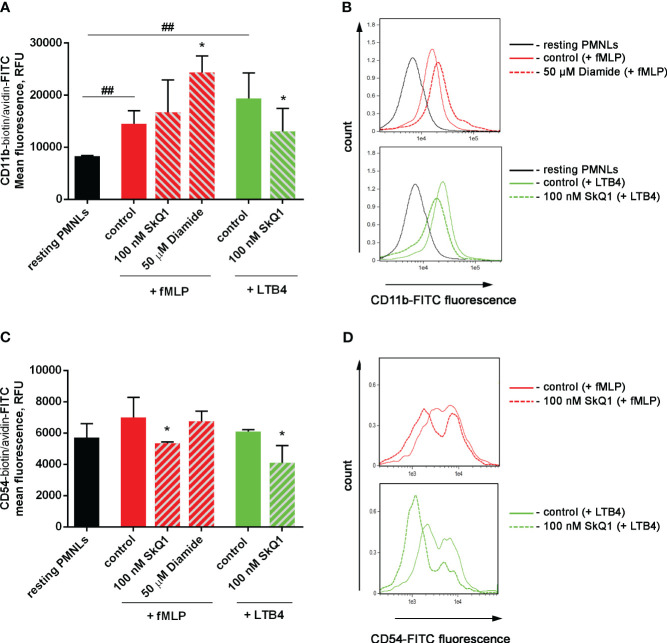
Effect of SkQ1 and diamide on adhesion molecules externalization upon (co-) stimulation with bacteria *S. typhimurium* and fMLP or LTB4. PMNLs (10^6^/mL HBSS/HEPES) were incubated for 20 min without additives (resting PMNLs), in the presence of bacteria (control) or bacteria in combination with 100 nM SkQ1 or 50 µM diamide (bacteria per cell ratio ~25:1). Then 0.1 µM fMLP (+ fMLP) or 0.1 µM LTB4 (+ LTB4) were added for 10 min followed by staining of surface CD11b and CD54 proteins and flow cytometry. Presented are the average values of three independent experiments (means ± SEM) **(A, C)** and typical histograms **(B, D)** of fluorescence for PMNLs stained with avidin-FITC in addition to biotynilated CD11b **(A, B)** and CD54 **(C, D)** antibodies. *p < 0.05 for pairs of data compared to corresponding control values; ^##^p < 0.01 for the specified data pairs as shown by one-way ANOVA with Dunnett’s multiple comparison test.

Activation of neutrophil adhesive properties can be reflected by adhesive receptor expression, first of all CD11b, a component of Mac-1 (CD11b/CD18) β2 integrin (58). In experiments with binding of fluorescently labelled antibodies to CD11b it was shown that both fMLP and LTB4 increased CD11b density on the surface of the cells, and diamide potentiated effect of fMLP. SkQ1 did not influence fMLP-induced expression, but decreased LTB4-induced effects (**Figures 4A, B**). The surface CD54 (ICAM-1) expression on neutrophils correlates with neutrophil migration, i.e. with cell-cell communication (59); and anti-CD54 monoclonal antibody inhibited neutrophil aggregation and formation of inter-cellular contacts (60). CD54 increased significantly on migrated PMNs; with rather low CD54 expression on adherent neutrophils (61). In our experimental model CD54 plays role as counter-receptor for integrins during homotypic adhesion; and SkQ1 decreased CD54 surface expression on neutrophils, as in the presence of the “first” chemoattractant fMLP, as well as at adding of the “second” chemoattractant LTB4 (**Figures 4C, D**). These data indicate that SkQ1 affects not only LTB4 synthesis but also LTB4-dependent signaling. The increased adhesiveness of neutrophils induced by LTB4 provided the possibility of forming tight intercellular contacts, which can support swarming and clustering around pathogens.

## 4 Discussion

Redox regulation plays an important role in the activation of 5-LOX and the regulation of leukotriene synthesis in neutrophils. Specifically, enzymatic activity requires the oxidation of Fe^2+^ to Fe^3+^ at the 5-LOX active site. Lipid hydroperoxides are involved in the activation of 5-LOX, at least in part through the oxidation of Fe^2+^ (62, 63). 5-LOX catalyzes the biosynthesis of leukotrienes using arachidonic acid (AA) as a substrate. Phospholipase A2, which produces AA from phospholipids, can be activated by ROS and lipid hydroperoxides (64), promoting the activation of leukotriene synthesis through oxidative processes. At the same time, excess hydrogen peroxide has been shown to inhibit 5-LOX activity in alveolar macrophages in parallel with ATP depletion (65).

Our previous work (22) using the mitochondria-targeted antioxidant SkQ1 demonstrated the important role of mitochondrial ROS in leukotriene synthesis induced by the Ca^2+^ ionophore A23187, the chemoattractant fMLP, and the opsonized yeast cell wall components zymosan. Here, we showed (**Figure 1**) that mitochondria-targeted antioxidants suppress fMLP-induced leukotriene synthesis in neutrophils exposed to *S. typhimurium*. We also showed that leukotriene synthesis is inhibited by the respiration inhibitor antimycin A and the oxidative phosphorylation uncoupler FCCP, presumably due to inhibition of mtROS production stimulated by voltage-dependent Ca^2+^ accumulation in mitochondria. Inhibition of respiration or dissipation of membrane potential prevents the synthesis of mitochondrial ATP, but measurements of ATP content did not reveal the effect of antimycin A or FCCP in infected neutrophils (**Figure 1B**).

Inhibition of glucose metabolism by 2-deoxy-D-glucose (2-DG) led to a decrease in ATP content and, in parallel to inhibition of leukotriene synthesis (**Figure 1C**) consistently with earlier data on the requirement of energy metabolism for the synthesis of leukotrienes (66). Glucose is catabolized by two fundamental pathways: glycolysis to produce ATP and the oxidative pentose phosphate pathway (PPP) to produce reduced nicotinamide adenine dinucleotide phosphate (NADPH). Very recently, it was shown that activation of the oxidative burst in neutrophils depends on a switch from glycolysis to a unique form of PPP called the “pentose cycle” (53). In this mode, all glucose-6-phosphate is consumed through PPP, while the initial steps of glycolysis are reversed to support pentose phosphates recycling. It has been proposed that this switch is required to maximize the supply of NADPH to fuel NADPH oxidase.

Another important NADPH-dependent enzyme is glutathione reductase, which reduce oxidized glutathione disulfide to sulfhydryl glutathione (67, 68). The main function of glutathione is the detoxification of electrophilic xenobiotics through condensation reactions catalyzed by glutathione S-transferases. Another protective function is mediated by the reduction of hydroperoxides catalyzed by glutathione peroxidase. This process appears to underlie the inhibition of 5-LOX by GSH observed in neutrophil homogenate (69). However, protein S-glutathionylation, activated by oxidative stress, may represent a more important regulatory mechanism.

The 5-lipoxygenase requires activation by fatty acid hydroperoxides (62). Hydroperoxides are inactivated in cells by GSH-dependent reduction by glutathione peroxidase, but diamide has the ability to non-enzymatically oxidize intracellular thiols (48). On this way diamide creates a demand for glutathione reduction by NADPH (70), and DPI provides high NADPH/NADP+ ratio (53) increasing stimulating effect of diamide (**Figures 2D, F**). The pentose phosphate pathway (PPP) is the major mechanism to maintain high NADPH/NADP+ ratio (71), and it was recently shown that PPP controls ROS production in crowding neutrophils (72). In accordance of the ability of diamide to switch glycolysis-dominant metabolism to pentose phosphate pathway (53), effects of diamide were less sensitive to glucose deprivation (**Figures 2G, H**). Effects of DPI were inhibited by glucose deprivation (**Figure 2G**).

Protein S-glutathionylation likely limits leukotriene synthesis in fMLP-activated neutrophils in the presence of *S. typhimurium*. This may be the reason that the GSH precursor NAC and the H_2_S donor sodium hydrosulfide, which increase the GSH/GSSG ratio, stimulate the synthesis of leukotrienes, in contrast to some other antioxidants (**Figure 2A**). In support of this proposal, we observed that thiol-oxidizing diamide (48), which oxidizes GSH, as detected by depletion of non-protein thiols (**Figure 2B**), at high concentrations (200-250 µM) significantly inhibits leukotriene synthesis (**Figure 2D**). This inhibition was effectively reversed by the GSH precursor SAMe (**Figure 2D**). Inhibition of leukotriene synthesis by FCCP or antimycin A may also be due in part to the GSH depletion caused by these agents (49–51). Accordingly, inhibition by FCCP and antimycin A was partially prevented by SAMe (**Figure 2E**). Importantly, diamide-induced thiol depletion was prevented by DPI (**Figure 2B**), indicating that inhibition of NADPH oxidase and subsequent decrease of oxidative stress and increase in NADPH levels may compensate for diamide-dependent GSH oxidation. Accordingly, DPI stimulated the synthesis of leukotrienes in the presence of diamide (**Figure 2D**). Inhibition of leukotriene synthesis by 2-DG-dependent ATP depletion may also be mediated by a decrease in GSH content. This may explain why the synthesis of leukotrienes in the presence of non-inhibitory doses of diamide, which is known to stimulate PPP-dependent NADPH production (52), was not inhibited by 2-DG in the presence of DPI (**Figure 2G**).

Neutrophils are the first cells in the foci of infection, and they have developed a set of mechanisms to turn on defense very quickly. To potentiate ROS production, they activate NADPH-oxidase, and NADPH is necessary for reduction of oxygen (73). To support NADPH-dependent ROS formation, neutrophils turn on PPP shunt (74). Activation of PPP is extremely important in supplying of NADPH (75). Inhibition of NADPH-oxidase shifted neutrophils from PPP cycle with ultra-high NADPH yield to glycolysis-dominant glucose metabolism (74). At the same time, neutrophil responses to pathogens include activation of glycolysis (76, 77).

Upregulation of PPP during oxidative stress contributes significantly to neutrophil responses, including not only oxidative burst (74) but also NETs formation (78). Redox regulation may involve various signaling pathways, including the MAP kinase-dependent pathway (79). We previously showed that activation of the MAP kinases Erk1/2 and p38, responsible for phosphorylation and activation of 5-LOX (46), is prevented by SkQ1 in neutrophils stimulated with A23187, fMLP, or opsonized zymosan (22). In neutrophils stimulated with *S. typhimurium* in combination with fMLP, the Erk1/2 inhibitor U0126 and the p38 inhibitor SB203580 inhibited leukotriene synthesis (**Figure 1E**). It is important to note that the GSH/GSSG ratio may also be involved in the regulation of Erk1/2, as S-glutathionylation, activated by oxidative stress, has been shown to play a key role in regulating MAP kinase kinase (MEKK1), that is an upstream kinase in the cascade of Erk1/2 activation (80). Using MS analysis, the authors demonstrated that oxidative stress induces glutathionylation of Cys1238 in the ATP-binding domain of MEKK1. This modification is easily reversible once the cell’s redox balance is restored. Thus, increasing the GSH/GSSG ratio with NAC or sodium hydrosulfide may improve Erk1/2 activation by preventing MEKK S-glutothionylation in neutrophils exposed to *S. typhimurium* and fMLP. Thus, redox regulation of Erk1/2 may be an important element in both mtROS-dependent and GSH-dependent regulation of leukotriene synthesis. Moreover, this regulation provides insight into why mitochondria-targeted antioxidants and NAC have opposing effects on leukotriene synthesis.

Our data (**Figure 3**) showed that *Salmonella typhimurium* in combination with fMLP stimulates the appearance of intercellular contacts in parallel with the synthesis of LTB4. The mitochondria-targeted antioxidant SkQ1 reduced this intercellular communication, as well as the synthesis of leukotrienes. Thus, during bacterial infection, redox processes in neutrophils play a decisive role in the synthesis of LTB4, ensuring neutrophil swarming - the influx of neutrophils to places of large microbial accumulations.

Excessive synthesis of leukotrienes and especially of LTB4 plays important role in pathogenesis of various inflammatory diseases (81–88). Mitochondria-targeted antioxidants, such as SkQ1, have been proposed as a promising therapy the same range of pathologies (89, 90). SkQ1 demonstrated strong anti-inflammatory activity in acute bacterial infection (91) and in the systemic inflammatory response syndrome (SIRS) model (92). The inhibition of leukotriene synthesis demonstrated above under conditions of pronounced activation of neutrophils can significantly contribute to the therapeutic effect of SkQ1. Importantly, our data indicate that administration of known thiol-based antioxidants such as NAC can dangerously stimulate leukotriene synthesis by neutrophils under the same conditions that mimic severe pathogenic infection.

## Data availability statement

The original contributions presented in the study are included in the article/**Supplementary Material**. Further inquiries can be directed to the corresponding authors.

## Ethics statement

The studies involving humans were approved by Experimental and the subject consent procedures were approved by the Bioethics Committee of the Lomonosov Moscow State University, Application # 6-h, version 3, Bioethics Commission meeting # 131-d held on 31.05.2021. The studies were conducted in accordance with the local legislation and institutional requirements. The participants provided their written informed consent to participate in this study.

## Author contributions

EG: Writing – original draft, Data curation, Formal analysis, Investigation, Methodology. GV: Investigation, Methodology, Writing – original draft, Funding acquisition. SG: Investigation, Methodology, Writing – original draft, Data curation. NK: Writing – review & editing, Methodology. TG: Conceptualization, Writing – review & editing. YR: Writing – review & editing, Methodology, Resources. KL: Writing – review & editing, Methodology. BC: Writing – review & editing, Conceptualization, Writing – original draft. GS: Conceptualization, Writing – original draft, Writing – review & editing, Project administration, Supervision.
