# Supplementary material for: Redox processes are major regulators of leukotriene synthesis in neutrophils exposed to bacteria Salmonella typhimurium; the way to manipulate neutrophil swarming
Source: Front Immunol. 2024 Feb 7;15:1295150. doi: 10.3389/fimmu.2024.1295150 (PMC10880102; doi:10.3389/fimmu.2024.1295150)
Supplement: Supplementary file 1 [file DataSheet_1.pdf]

Supplementary Material

Supplementary Figures

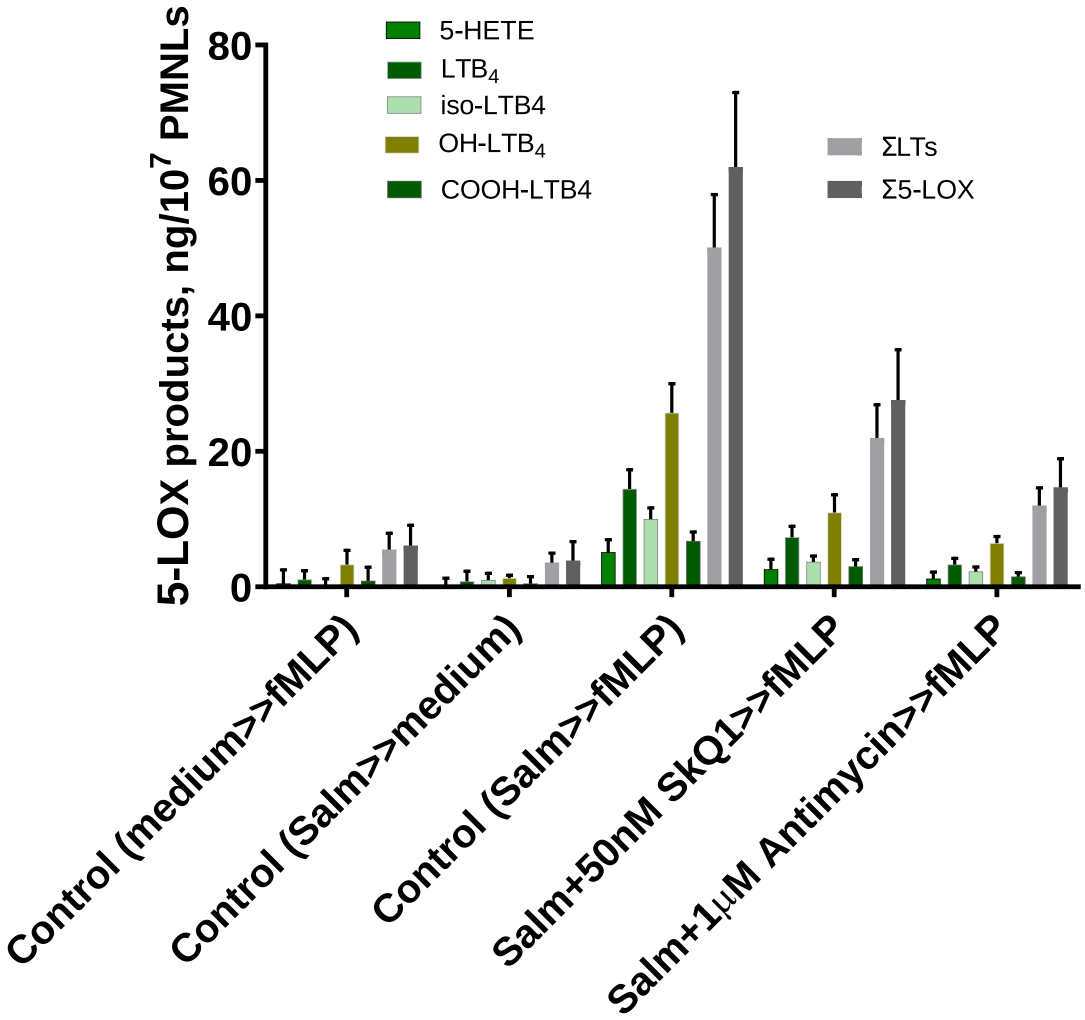

Supplementary Figure 1. 5-LOX product synthesis in human neutrophil (PMNLs samples (1.2–1.5) × 10^7/6 ml HBSS/HEPES). PMNLs incubated without bacteria (Control (medium>>fMLP)) or with bacteria *Salmonella typhimurium* (Salm) (the ratio of bacteria:PMNLs ~ 25:1) without (control) or with reagents indicated on x-axis for 30 min, followed by fMLP (0.1 µM) or solvent (Control (Salm>>medium)) addition for 10 min. The 5-LOX products were analyzed using HPLC, and data for 5-HETE, LTB_4, iso-LTB_4, ω-OH-LTB_4 and ω-COOH-LTB_4 are presented. ΣLTs= LTB_4, iso-LTB_4, ω-OH-LTB_4. Σ5-LOX=5-HETE, LTB_4, iso-LTB_4, ω-OH-LTB_4 and ω-COOH-LTB_4. Values indicate mean ± SEM of three independent experiments performed in duplicate.

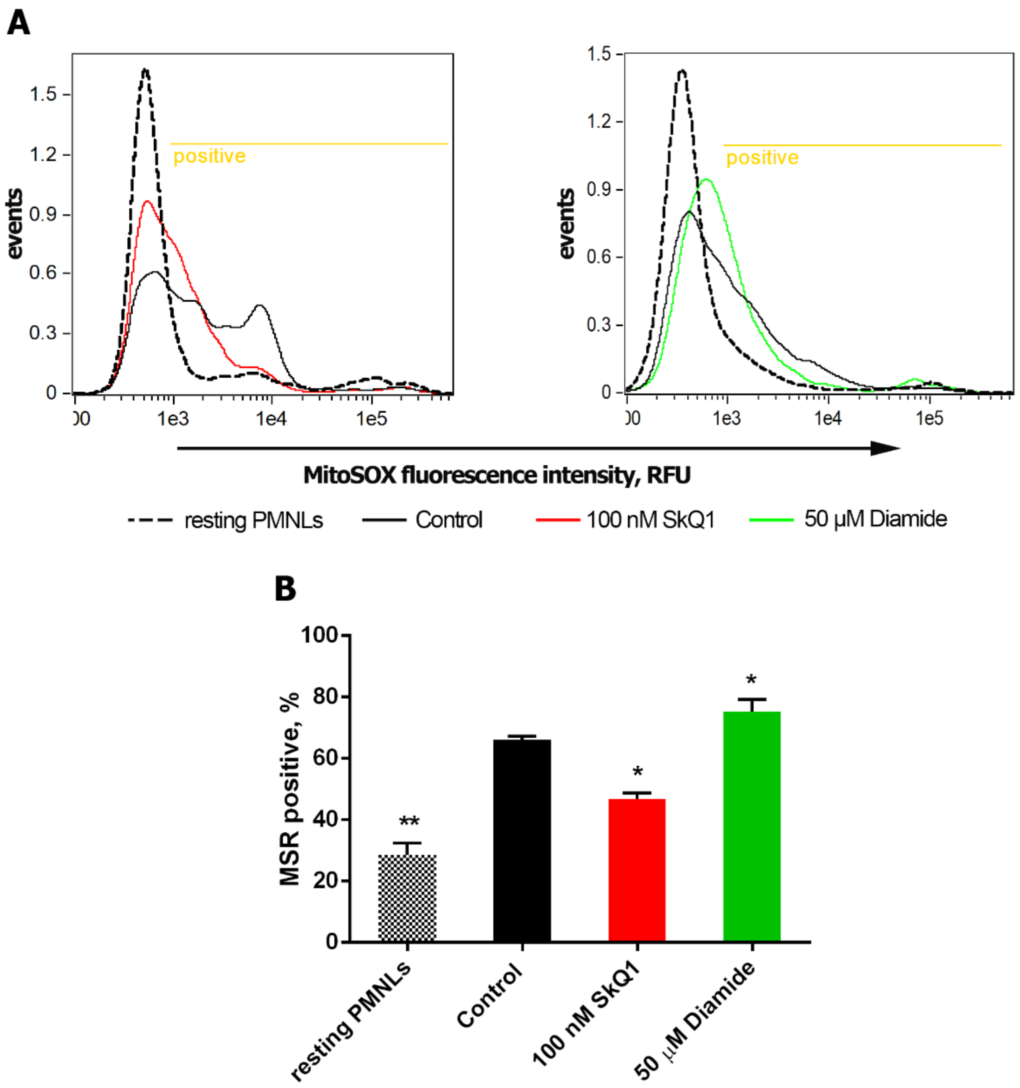

Supplementary Figure 2. SkQ1 and diamide effects on mitochondrial superoxide production in stimulated neutrophils. PMNLs (10^6 cells/probe) were suspended in HBSS/HEPES, kept for 5 minutes, after which MitoSOX was added to a final concentration of 1 µM. Then *S. typhimurium* (bacteria per cell ratio ~25:1) and indicated reagents were added for 20 min followed by 0,1 µM fMLP stimulation for 7 min. Resting PMNLs samples were incubated without any additives. Treated cells were pelleted by centrifugation for 10 min at 200 g, 4 °C and analyzed by flow cytometry 610/30 nm spectral region on Amnis FlowSight Imaging Flow Cytometer (Luminex Corp., Austin, Texas, USA). IDEAS Image Data Exploration and Analysis Software (Luminex Corp., Austin, Texas, USA) was used for data analyzing. Presented are typical fluorescence histograms (A) and data of mean ± SEM of MitoSOX Red (MSR) positive PMNLs subpopulation. *$p < 0.05$; **$p<0.01$ compared to Control sample as shown by one-way ANOVA with Dunnett's multiple comparison test.

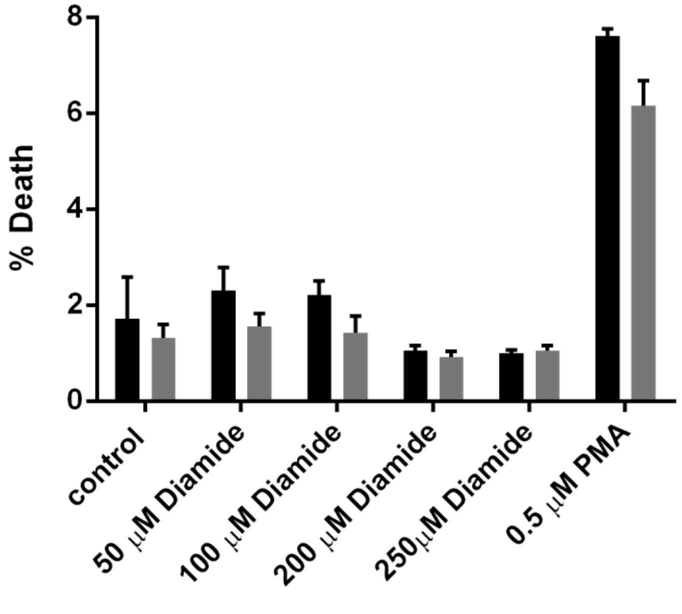

Supplementary Figure 3. Cytotoxicity was quantified by detecting cells with compromised plasma membrane using propidium iodide (PI). PMNLs were seeded into the wells of a 96-well plate (5×10^5 cells/well) with a pre-warmed HBSS/HEPES containing PI dye (50 μg/mL). After 5 minutes, fluorescence at ex/em 544/612 nm was measured, intensity average value obtained was taken as minimal (no more than 1% of dead cells, which was confirmed by microscopy of a sample stained with Trypan blue dye). Cells were then incubated in the absence (control) or presence of diamide (concentration range 50–250 μM) alone (black bars) or under the experimental conditions used in the study, namely 20 minutes of incubation in the presence of bacteria or bacteria with diamide followed by the addition of 0.1 μM fMLP (grey bars). 0.5 μM PMA was used as a positive control. Fluorescence was measured every 10 minutes for 2 hours from the moment of diamide adding. Presented are the average values ±SEM of the proportion of dead cells (%), obtained by normalizing the detected fluorescence intensities (2 hours endpoint) to the difference between the maximum (100 % membrane integrity loss that detected after adding 0.02 % Triton X100) and minimum fluorescence intensities.

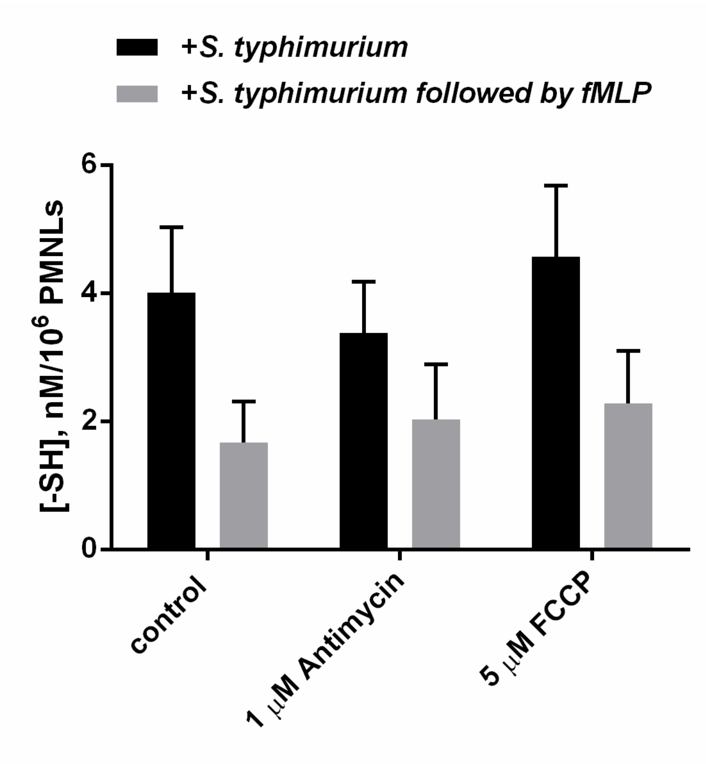

Supplementary Figure 4. Antimycin A and FCCP effects on intracellular reduced thiols level in neutrophils. PMNLs were pre-incubated for 10 min, then *S. typhimurium* alone (Control) or bacteria together with indicated stimuli were added for 20 min (black bars) followed by 0.1 μM fMLP treatment for 5 min (grey bars). After the exposure time, the cells were pelleted while cooling and assayed for reduced -SH content with Ellman's reagent. Values are given as mean ± SEM of the content of reduced thiols in the samples, nM per 10^6 PMNLs.

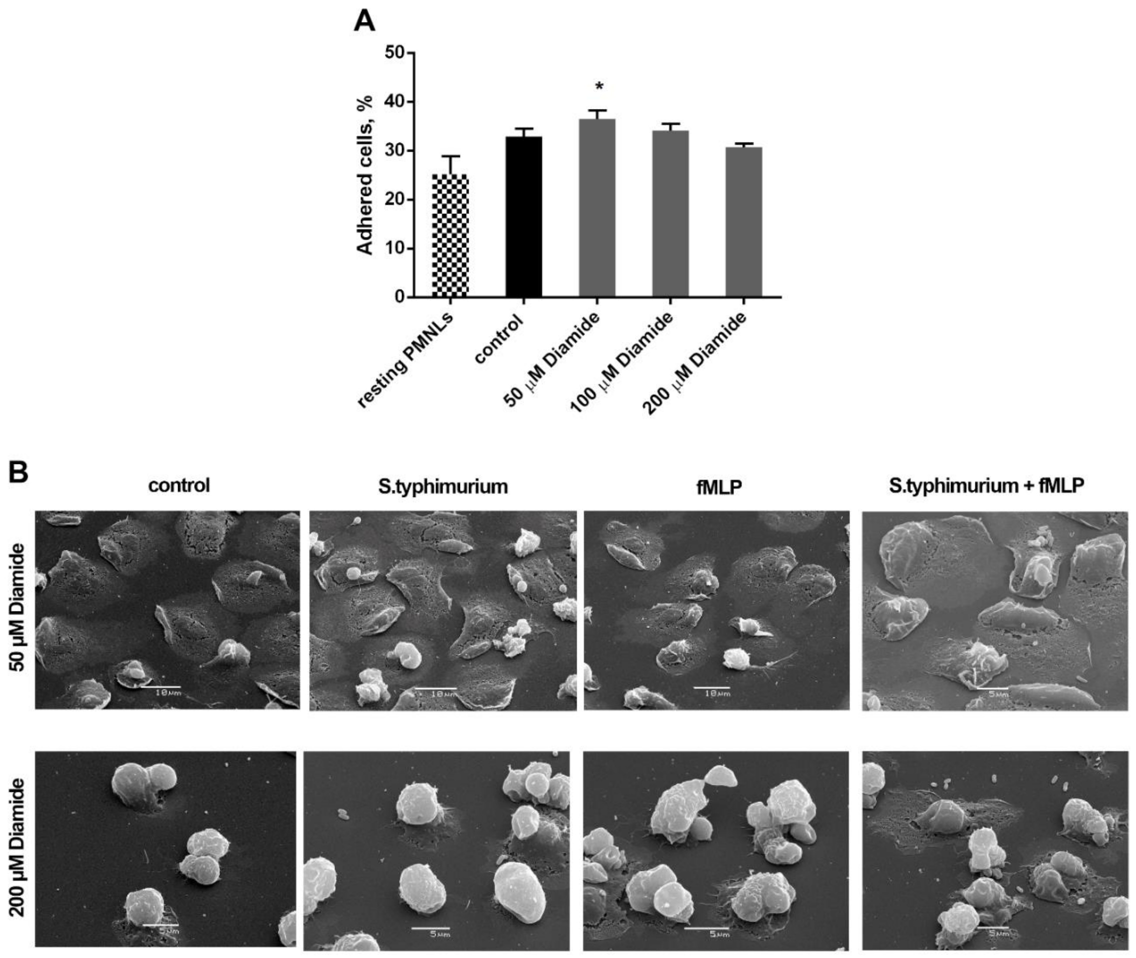

Supplementary Figure 5. Effect of diamide on neutrophil adhesiveness (**A**) and morphology (**B**) upon (co-)stimulation with bacteria *S. typhimurium* and fMLP. **A.** PMNLs (2×10^5 cells/sample) were seeded onto fibrinogen-coated 96-well plates containing pre-warmed HBSS/HEPES without additives (resting PMNLs), with *S. typhimurium* (control) or *S. typhimurium* supplemented with indicated concentrations of diamide (bacteria per cell ratio ~20:1). After 20 min incubation at 37°C, 5% CO_2, 0.1 μM fMLP was added to all wells, with the exception of the resting PMNLs sample. Cells were incubated for another 10min, after which the unattached cells were removed by carefully washing. 4 mM H_2O_2 and permeabilizing buffer (67 mM Na_2HPO_4, 35 mM citric acid and 0.1% Triton X-100) containing 5.5 mM o-phenylenediamine dihydrochloride (OPD) were added for 5 min. The reaction was stopped with 1 M H_2SO_4. The percentage of attached neutrophils was determined by measuring the absorption (490 nm) of 2,3-diaminophenazine, a product of the myeloperoxidase-catalyzed OPD oxidation with H_2O_2, and comparing the obtained values with the calibration ones. Values shown are means ± SEM of three independent experiments performed in triplicates; $*p < 0.05$ compared to control sample as shown by one-way ANOVA with Dunnett's multiple comparison test. **B.** PMNLs (10^6/ml HBSS/HEPES) were plated on coverslips in culture dishes and, after 10 min pre-incubation, they were cultured for 20 min in the presence of 50 μM diamide, 200 μM diamide without additional stimulation (**control**) or with the addition of bacteria (bacteria per cell ratio ~25:1) (**S. typhimurium**). After this, fMLP (0.1 μM sample concentration) was added to the samples for 5 minutes (**fMLP**; and **S. typhimurium+fMLP**). At the end of each stage, PMNLs samples were fixed and subsequently visualized using scanning electron microscopy.
